# Transcriptome analysis of sexual dimorphism in dorsal down coloration in goslings

**DOI:** 10.1186/s12864-024-10394-z

**Published:** 2024-05-22

**Authors:** Yi Liu, Guangquan Li, Zhanbao Guo, Huiling Zhang, Baozhi Wei, Daqian He

**Affiliations:** 1 Shanghai Academy of Agricultural Sciences, Institute of Animal Husbandry and Veterinary Science, Shanghai, China; 2 Chinese Academy of Agricultural Sciences, Institute of Animal Sciences, Beijing, China; 3 Shandong Rongda Agricultural Development Co., Ltd, Shandong, China

**Keywords:** Sexual Dimorphism, Autosexing, Transcriptome Analysis, Goose

## Abstract

**Background:**

In day-old Hungarian white goose goslings, there is a noticeable difference in dorsal down coloration between males and females, with females having darker dorsal plumage and males having lighter plumage. The ability to autosex day-old goslings based on their dorsal down coloration is important for managing them efficiently and planning their nutrition in the poultry industry. The aim of this study was to determine the biological and genetic factors underlying this difference in dorsal down colorationthrough histological analysis, biochemical assays, transcriptomic profiling, and q‒PCR analysis.

**Results:**

Tissue analysis and biochemical assays revealed that compared with males, 17-day-old embryos and day-old goslings of female geese exhibited a greater density of melanin-containing feather follicles and a greater melanin concentration in these follicles during development. Both female and male goslings had lower melanin concentrations in their dorsal skin compared to 17-day-old embryos. Transcriptome analysis identified a set of differentially expressed genes (DEGs) (*MC1R*, *TYR*, *TYRP1*, *DCT* and *MITF*) associated with melanogenesis pathways that were downregulated or silenced specifically in the dorsal skin of day-old goslings compared to 17-day-old embryos, affecting melanin synthesis in feather follicles. Additionally, two key genes (*MC1R* and *MITF*) associated with feather coloration showed differences between males and females, with females having higher expression levels correlated with increased melanin synthesis and darker plumage.

**Conclusion:**

The expression of multiple melanogenesis genes determines melanin synthesis in goose feather follicles. The dorsal down coloration of day-old Hungarian white goose goslings shows sexual dimorphism, likely due to differences in the expression of the *MC1R* and *MITF* genes between males and females. These results could help us better understand why male and female goslings exhibit different plumage patterns.

## Background

Sexual dimorphism in plumage coloration is common among avian species [1]. Among Hungarian white geese, this dimorphism is evident in the dorsal down coloration of newly hatched goslings, with females having dark grey plumage and males displaying lighter shades of grey or light yellow. The sex determination of day-old goslings can be largely performed by observing differences in dorsal down characteristics, although this method may not be 100% accurate [2]. Compared with vent sexing, utilizing differences in feather phenotypes for automatic sex identification is cost-effective and efficient, providing faster results. Most commercial egg–laying strains can be sexed by their wing feathers at the day-old stage. However, color sexing cannot be applied to most goose breeds (both females and males are born with yellow fluff), except for the Hungarian white goose. Therefore, the Hungarian white goose serves as an ideal model for studying sexual dimorphism in goose plumage coloration and could be an essential breeding material for generating additional autosexing strains based on feather phenotypes.

Animal coloration is primarily determined by pigments, which play a crucial role in the vast array of coloration observed in the animal kingdom. Numerous studies have consistently demonstrated that color morphs in a wide range of bird species are primarily attributed to melanins and carotenoids, with melanins being more prevalent [3, 4]. Although the genetic mechanisms underlying carotenoid-based coloration remain largely unexplored, melanin-based color traits offer a valuable model system for investigating the genetic basis of phenotypic diversity [5]. Melanin, the most prevalent pigment in animal coloration, contributes to the vibrant feather colors observed in birds. Avian melanin pigmentation often shows widespread sexual dimorphism [6]. Therefore, our study focused on investigating the sexual dimorphism of melanin-based plumage coloration in goslings.

Bird plumage encompasses two main types of melanin: eumelanin, which produces dark black, brown, or grey colors, and pheomelanin, which creates lighter yellowish to reddish hues [7]. Typically, avian feathers contain a blend of these melanins. Variations in melanin-based color traits primarily arise from differences in the quantity, proportion, and distribution of eumelanin and pheomelanin, as well as through processes such as melanoblast migration, differentiation, and melanosome structure and transport [5]. In avian species, white and yellow plumage lack detectable levels of eumelanin due to the absence of eumelanosomes required for its synthesis [8]. These findings suggest that the chemical composition and concentration of melanin play a crucial role in determining feather coloration in birds. Day-old goslings exhibit three distinct colors, namely pale yellow, light gray, and black with evident sexual dimorphism. However, the underlying mechanisms responsible for the development of sexually dimorphic plumage coloration remain poorly understood and necessitate further investigation into the interplay between eumelanin and pheomelanin production in goslings.

Melanin synthesis is mediated by melanosomes located within melanocytes and is influenced by various extracellular factors, including specific enzymes and other crucial regulatory and structural proteins. Tyrosinase and dopachrome tautomerase are the two most studied enzymes involved in melanogenesis. Tyrosinase, often considered the rate-limiting enzyme, is indispensable for melanin biosynthesis within melanocytes [5]. The genetic regulation of avian melanin biosynthesis involves the expression of key enzymes responsible for melanin synthesis, along with other vital regulatory factors [7]. Existing research has sought to elucidate associations between variations in genotype and gene expression levels with phenotypic differences in melanin-based coloration. Consequently, more than 50 genes linked to plumage coloration have been identified in avian species [8]. Several of these genes, including the melanocortin receptor 1 (*MC1R*) gene [9–11], tyrosinase (*TYR*) gene [11, 12], tyrosinase-related protein 1 (*TYRP1*) gene [13], endothelin receptor B2 (*EDNRB2*) gene [14], dopachrome tautomerase (*DCT*) gene [15], and microphthalmia-associated transcription factor (*MITF*) [16] gene, among others, have been extensively investigated for their role in modulating melanogenesis, melanoblast migration, and melanocyte differentiation, thereby influencing melanin-based plumage coloration. Moreover, comprehensive studies have investigated how different variants within these candidate genes interact with feather color in various duck populations and breeds [17–22]. Additionally, *MITF* serves as a pivotal switch gene that regulates the majority of downstream genes involved in the melanogenesis pathway [16]. The Wnt, cAMP, and MAPK signaling pathways play important roles in regulating the cycle of melanocyte the development and maintenance of hair follicles, the synthesis and transport of melanin, and the pigmentation of hair and skin. MITF is central to these processes. Despite extensive research on the synthesis mechanism of melanin deposition in various feather color types, the molecular mechanisms underlying sexual dimorphism in plumage coloration between male and female geese remain poorly understood. This study aimed to compare the levels of melanin in the skin on the backs of male and female geese, determine where melanin is found in their feathers, and analyze the genes involved in sexually dimorphic plumage coloration based on melanin. The results should provide a better understanding of the mechanisms governing sexual dimorphism in gosling plumage formation.

## Materials and methods

### Animal ethics

The animals in this study were approved and followedthe 'Regulations on the Administration of Laboratory Animals' promulgated by the State Council of the People's Republic of China in 2017. This study was authorized by the Animal Ethics Committee of Shanghai Academy of Agricultural Sciences (Shanghai, China) under approval number (SAASPZ0522046). We conducted this research following the ARRIVE guidelines (https://arriveguidelines.org) and in accordance with ethical guidelines and regulations for animal treatment.

### Animals and sample collection

**Fig. 1:**
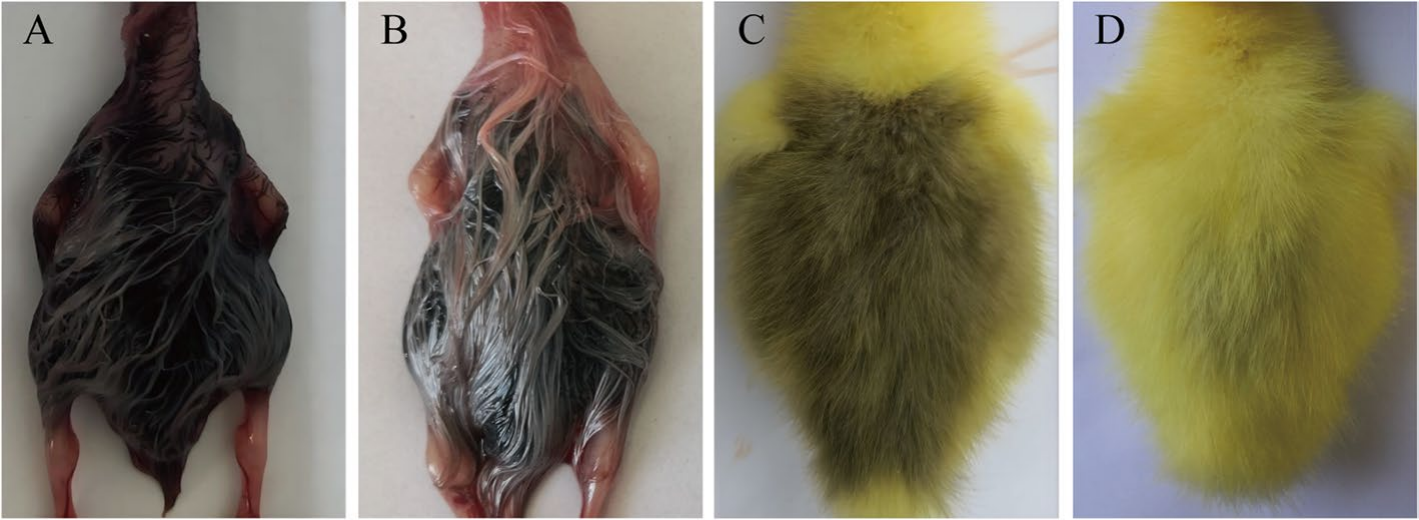
Dorsal down coloration in goslings at the 17-day embryo and day-old stages. **A** and **B** depict the dorsal down coloration of female and male geese, respectively, in 17-day-old embryos. **C** and **D** show the dorsal down coloration of female and male goslings at the day-old stage, respectively

All skin tissue samples were collected in strict adherence to animal welfare regulations. The Hungarian white goslings selected for the experiment were euthanized via carbon dioxide inhalation, followed by cervical dislocation by trained individuals. The samples from E17 (embryos at 17 days of age) (*n* = 12, 6 females and 6 males) and D0 (newly hatched goslings) (*n* = 12, 6 females and 6 males) were obtained from the Zhuanghang Research Farm, affiliated with the Shanghai Academy of Agricultural Sciences, Shanghai, China. The dorsal down coloration of goslings at the E17 and D0 stages is depicted in Fig. 1. After euthanasia, the feathers were removed to preserve intact feather follicle tissue. Skin tissue devoid of subcutaneous adipose tissue was collected and processed in triplicate after gentle washing with phosphate-buffered saline (PBS, Cyclone, Logan, UT, USA). These tissue sections were fixed in 4% paraformaldehyde at room temperature. Simultaneously, samples for ELISA analysis were immediately stored at -80 °C. The remaining samples intended for RNA extraction were subsequently flash-frozen in liquid nitrogen and stored at -80 °C.

### Histological analysis

Skin tissues were removed from the fixative and processed through dehydration, wax infiltration, embedding, and sectioning at a thickness of 4 µM for subsequent silver nitrate staining. The paraffin sections were then sequentially immersed in xylene I for 20 min, followed by xylene II for another 20 min. They were subsequently treated with anhydrous ethanol I and II for 5 min each, followed by immersion in 75% ethyl alcohol for an additional 5 min. The sections were then rinsed thoroughly with tap water and distilled water through a series of 3–5 washes. Each slide was stained using a Masson Fontana Stain Kit (Wuhan Servicebio Technology Co., Ltd., Wuhan, China) and sealed with a neutral resin. The prepared skin tissue sections were examined under a Nikon Eclipse E100 microscope (Nikon, Tokyo, Japan), and images were acquired using the NIKONDS-U3 imaging system (Nikon, Tokyo, Japan).

### ELISA Assay

The melanin content in the skin tissues was quantified using ELISA. Goose skin tissue weighing 0.1 g was transferred to a 2 mL EP tube, followed by the addition of 1 mL of normal saline. The mixture was thoroughly homogenized and then centrifuged at 3000 RPM for 10 min to obtain the supernatant. Melanin concentrations in the skin tissues were quantified using an enzyme-linked immunosorbent assay (ELISA) kit for goose melanin obtained from Beijing JINZHIYAN Biotechnology Co., Ltd., Beijing, China, following the manufacturer's instructions.

### Total RNA isolation and Illumina sequencing

Skin tissues were subjected to RNA extraction using TRIzol reagent (Invitrogen, Carlsbad, CA, USA) following the manufacturer's protocol. The concentration, quality, and integrity of the RNA were assessed using a NanoDrop spectrophotometer (Thermo Fisher Scientific Inc., Waltham-, MA, USA). The cDNA libraries were prepared using the NEBNext Ultra II RNA Library Prep Kit for Illumina (New England Biolabs Inc., Ipswich, Massachusetts, USA) from 3 μg of high-quality total RNA (OD260/280 > 2.0, OD260/230 > 2.0) following the established protocol. Thereafter, cDNA libraries underwent shearing, purification, blunting of ends, and ligation to adapters optimized for Illumina sequencing. Library products with 400–500 base pairs were enriched and purified using the AMPure XP system (Beckman Coulter, Beverly, CA, USA). DNA fragments with ligated adaptor molecules on both ends were selectively amplified using Illumina PCR Primer Cocktail in a 15-cycle PCR. The resulting products were purified again using the AMPure XP system and quantified by Agilent high sensitivity DNA assay on a Bioanalyzer 2100 system (Agilent Technologies, Santa Clara, CA, USA). Finally, each sequencing library was sequenced on the NovaSeq 6000 platform (Illumina, USA) at Shanghai Personal Biotechnology Co., Ltd., China.

### Transcriptome analysis

To obtain high-quality sequences, fastp (version 0.22.0) software was used to eliminate connectors and low-quality reads from the raw data. The filtered reads were subsequently aligned to the reference genome using HISAT2 (version 2.1.0) [23]. For gene expression analysis, HTSeq (version 0.9.1) [24] was used to count the Read Count values for each gene, which were then normalized to the FPKM values. Differential expression analysis was performed using DESeq (version 1.38.3) [25], applying the following filter criteria: an absolute |log2FoldChange|> 1 and a significant *P* value < 0.05, and Benjamini–Hochberg false discovery rate (FDR) was used to correct the *P* value [26]. We employed the R language Pheatmap (version 1.0.12) [27] software package to perform bidirectional clustering analysis of DEGs in the samples. A heatmap was generated based on the expression levels of the same gene across different samples and the expression patterns of different genes within the same sample using Euclidean distance calculations and the complete linkage method for clustering.

Moreover, GO and KEGG pathway enrichment analyses of the DEGs were carried out. GO enrichment analysis for functional significance utilized the hypergeometric test to map all differentially expressed genes (all DEGs/upregulated DEGs/downregulated DEGs) to terms in the Gene Ontology database using topGO (version 2.50.0) [28]. We searched for significantly enriched GO terms and calculated P-values using the hypergeometric distribution method (the standard for significant enrichment was a *P* value < 0.05). ClusterProfiler (version 4.6.0) [29] software was used to carry out the enrichment analysis of KEGG pathways for differentially expressed genes, focusing on pathways significantly enriched with a *P* value < 0.05.

### Validation of DEGs results using qRT‒PCR

To validate the differential expression of genes associated with melanogenesis by transcriptome analysis, we selected five DEGs for quantitative real-time PCR (qRT‒PCR) analysis. According to the previously assembled sequences, the primers for six candidate genes were designed using Oligo 6.0 software. Information about the six candidate genes and their corresponding primers is provided in Table 1. The qRT‒PCR was performed using the SYBR Premix Ex TaqTM II Reagent Kit (RR820A, Takara, Dalian, China) and an Applied Biosystems 7500 Fast Real-Time PCR System (7500, ABI, USA). The qRT‒PCR mixture consisted of 1 µL of cDNA template, 10 µL of SYBR Premix ExTaq, 0.4 µL of Rox Reference Dye(II), 7.4 µL of nuclease-free water, and 0.6 µL of each gene-specific primer. The amplification programs followed the established protocol, including an initial denaturation step at 95 °C for 2 min, followed by 40 cycles of denaturation at 95 °C for 30 s, annealing at 58 °C for 30 s, and extension at 72 °C for 30 s. Subsequently, a thermal denaturing step was performed to generate melt curves. The relative expression levels of the selected candidate genes were quantified based on the threshold cycle value (Ct) and normalized to that of GAPDH using the equation ΔΔCt = Ct (target gene)—Ct (GAPDH), where ΔΔCt represents the fold change.

**Table 1:** Real-time PCR primer sequences

Gene Name	Primer SequenCes (5’ –3’)	Annealing Temperature	Size of target fragments
MITF	F:AGCTCGGGCACATGGACT R:AGAGAGGGTATCGTCCATCA	65℃	280bp
TYRP1	F:AATGAGATGTTTGTTACTG R:ACTGATCAGTGAGAAGAGG	65℃	208bp
TYR	F:GCGACTGAGAACGAGAAGAA R:AAGAGTGTGTCCCGAGAGGC	56℃	222bp
DCT	F:CCGCAATTCCAGTTTCAGCT R:ACCGCTTCATCCACTCATCA	56℃	209bp
MC1R	F:CAAGACGCTCTTCATGCTGC R:ATGGTGATGTAGCGGTCCAC	65℃	159bp
GAPDH	F:GGTGGTGCTAAGCGTGTCAT R:CCCTCCACAATGCCAAAGTT	60℃	200bp

The candidate genes: *MITF* Microphthalmia-associated transcription factor, *TYRP1* Tyrosinase-related protein 1, *TYR* Tyrosinase, *DCT* Dopachrome tautomerase, *MC1R* Melanocortin receptor 1 and *GAPDH* Glyceraldehyde-3-phosphate dehydrogenase

F denotes forward primers and R denotes reverse primers

### Statistical analysis

Data management and analysis of melanin levels and expression levels of candidate genes were conducted using Excel 2007. Statistical analysis was performed using one-way ANOVA in SPSS 26.0 software, followed by the Duncan's multiple comparison test. A level of *P* < 0.05 was used to determine statistically significant differences.

## Results

### Histological analysis of dorsal skin tissues

**Fig. 2:**
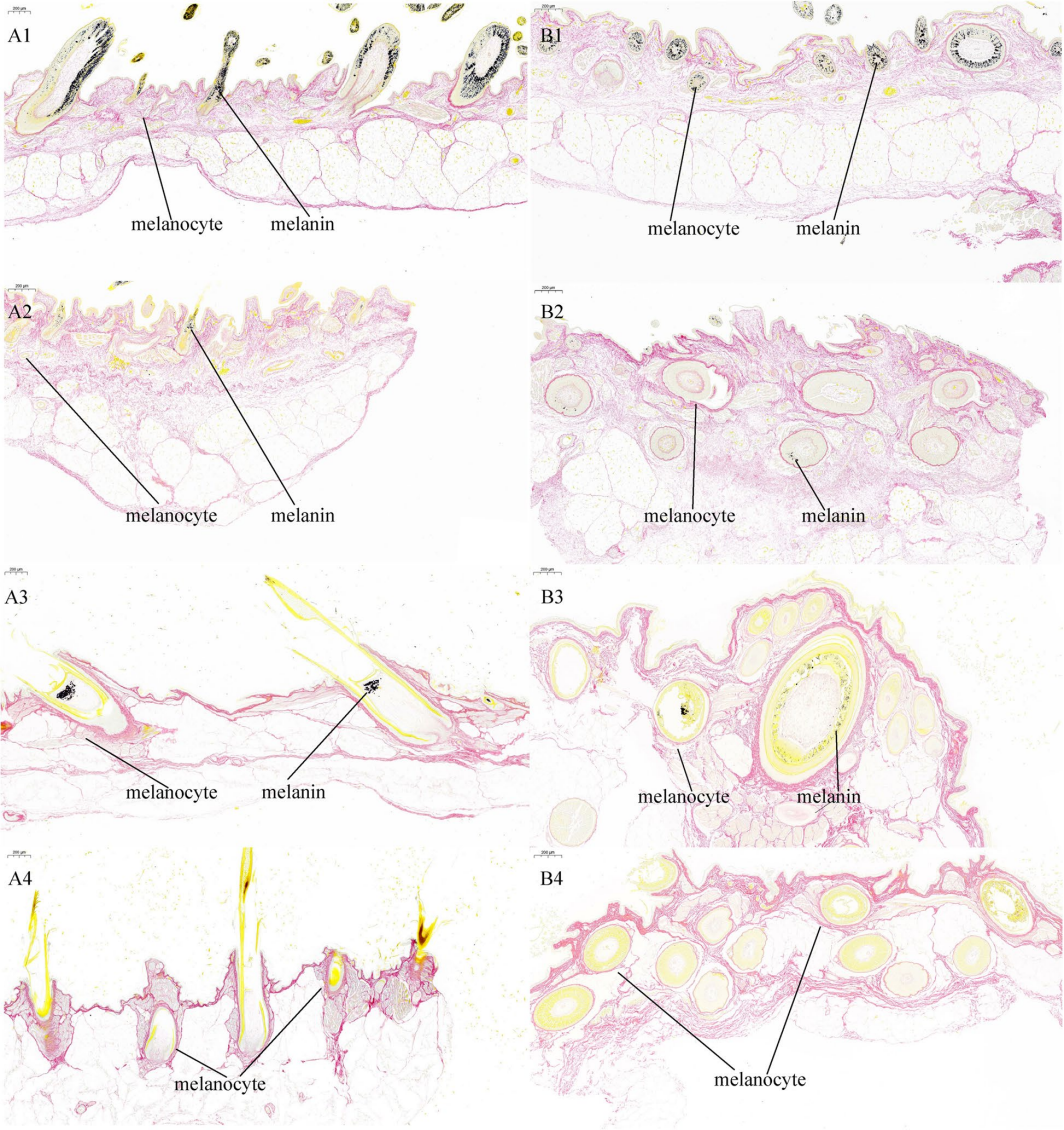
Histological examination of dorsal skin tissues in goslings at two distinct developmental stages. Representative images of silver nitrate-stained skin sections were acquired through vertical slitting (A1-A4) and horizontal slitting (B1-B4), with a scale of 200 μm. The melanin distribution in 17-day-old female goose embryos (A1 and B1) and male goose embryos (A2 and B2) was examined. The melanin distribution in day-old female goslings (A3 and B3) and male goslings (A4 and B4) was examined. The melanocytes and melaninare marked with lines on the histological images

We examined the dorsal skin tissues of goslings at two developmental stages, E17 and D0 goslings, determine whether if there were differences in melanin quantity and distribution within feather follicles between male and female goslings. We used silver nitrate staining to stain skin sections for examination. The images in Fig. 2 show the results of these examinations. The results reveal distinct differences in melanin distribution within skin feather follicles. At the E17 development stage, female geese had more melanin-containing feather follicles with more melanin (Fig. 2 A1 and B1) than male geese (Fig. 2 A2 and B2). However, the amount of melanin in the feather follicles of female goslings was significantly lower in D0 goslings (Fig. 2 A3 and B3) than in E17. In D0 goslings, melanin was concentrated at the central position of the feather roots (Fig. 2 A3), while it was evenly distributed primarily on the surface of the feather bud (Fig. 2 A1) of E17. However, melanin was not detected in the feather follicles of E17 male goslings (Fig. 2 A4 and B4).

### Determination of melanin content in dorsal skin tissues

**Fig. 3:**
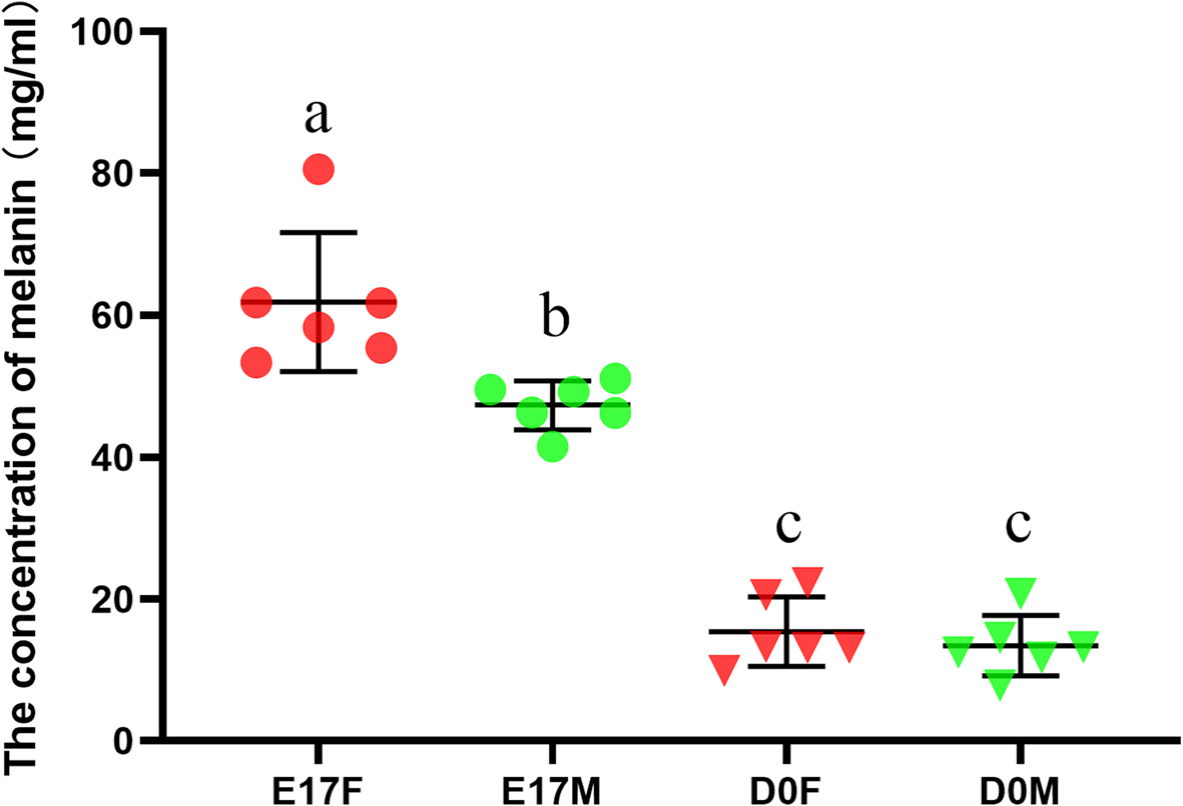
Concentrations of melanin in dorsal skin tissues of goslings at two distinct developmental stages. (E17F: 17-day-old female goose embryos, E17M: 17-day-old male goose embryos, D0F: day-old female goslings, D0M: day-old male goslings, *n* = 6; Different letters represent significant differences (*p* < 0.05)

The melanin content in the dorsal skin of goslings at two developmental stages was quantitatively assessed using ELISA, and a comparison of sexual dimorphism was conducted. At the E17 stage, the melanin content in the dorsal skin of female goslings was significantly greater than that in the dorsal skin of male goslings (Fig. 3). However, there were no differences between females and males at the D0 stage (Fig. 3). Compared to those at the E17 stage, both female and male individuals exhibited a significant reduction in melanin concentrations in the dorsal skin at the D0 stage (Fig. 3).

### Analysis of differentially expressed genes in dorsal skin

**Fig. 4:**
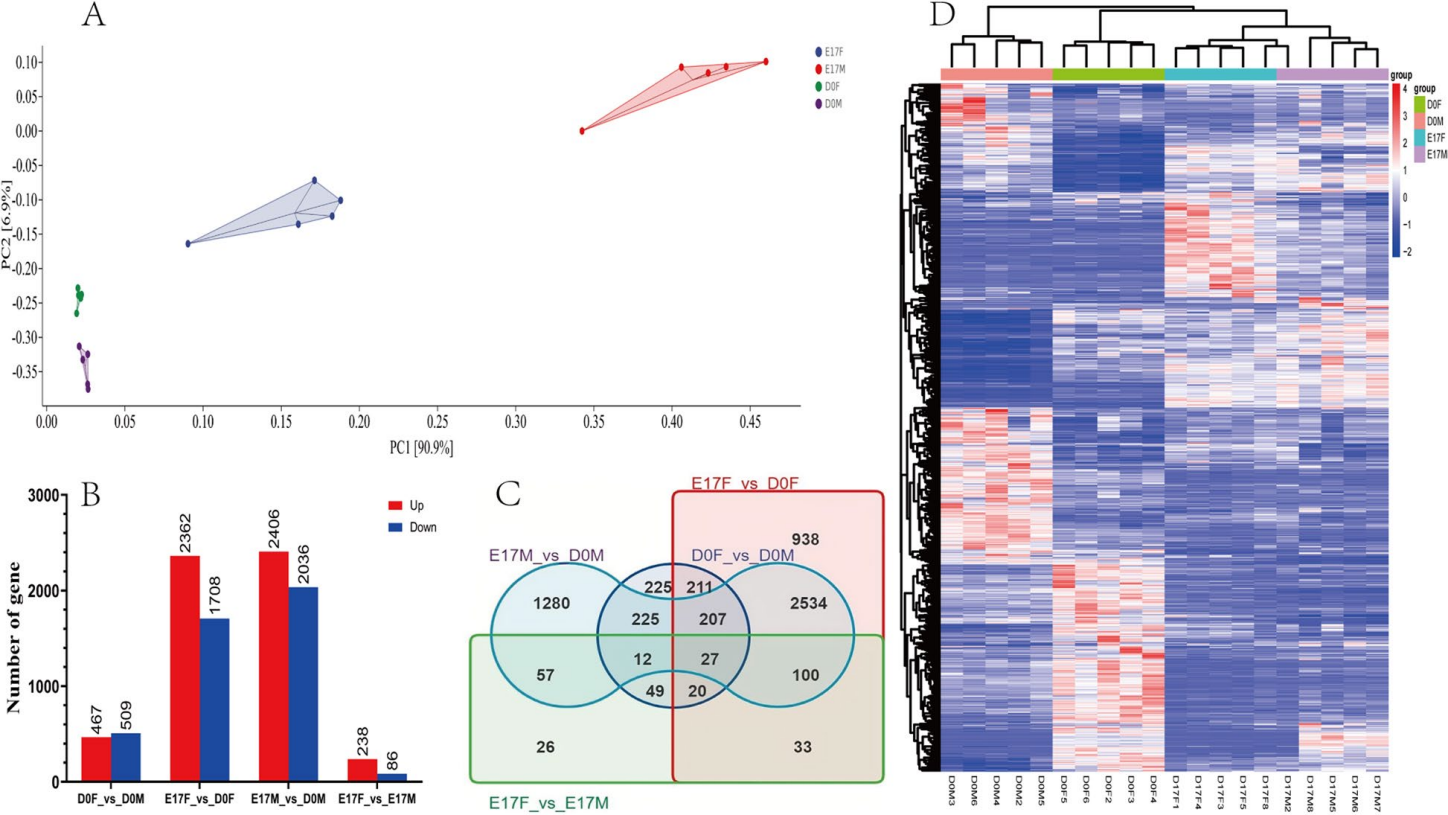
Transcriptome sequencing overview, including (**A**) principal component analysis conducted for each mRNA-Seq sample, (**B**) identification of differentially upregulated and downregulated genes in each group, (**C**) intersection analysis of differentially expressed genes between groups performed using a Venn diagram, and (**D**) visualization of differential gene expression (DGE) through a heatmap depicting higher expression levels in shades of red and lower expression levels in shades of steel blue. Additionally, upregulated genes are color-coded in red, and downregulated genes are color-coded in steel blue, as indicated by the accompanying color bar

Dorsal skin samples were collected at two distinct developmental stages, E17 and D0 goslings, for mRNA-Seq analysis. In total, 20 samples were obtained and all raw data have been deposited in the SRA database (accession number PRJNA1039165). Principal Component Analysis (PCA) results revealed four major clustering patterns among samples from different developmental stages and sexes, with samples from the same developmental stage and sexes exhibiting similar characteristics (Fig. 4A). Differential expression genes (DEGs) analysis based on mRNA-Seq data enabled the investigation of sexual dimorphism at distinct developmental stages through pairwise comparisons, with a significance threshold of |log2-fold change|> 1 and P-adjust < 0.05. There were 324 genes whose expression significantly differed between the sexes in the dorsal skin of the embryos at the E17 stage, with 238 transcripts upregulated and 86 transcripts downregulated in females compared to males (Fig. 4B). In D0 goslings, a total of 976 differentially expressed transcripts were identified in the dorsal skin when comparing females and males, with 509 transcripts upregulated in males and 467 transcripts upregulated in females (Fig. 4B). Compared to those in E17 and D0 goslings, 4068 DEGs were detected in female geese, while 4442 DEGs were detected in male geese (Fig. 4B). We then overlapped the list of DEGs between groups using a Venn diagram (Fig. 4C). The Venn diagram shows the DEGs in the dorsal skin between male and female individuals during the developmental stages of E17 and D0. A total of 5,944 DEGs were identified. Specifically, there were 324, 976, 4068, and 4442 DEGs in the comparisons of E17F vs E17M, D0F vs D0M, E17F vs D0F, and E17M vs D0M, respectively. Among the DEGs, 108 common DEGs were identified between the E17F vs E17M and D0F vs D0M libraries and were predominantly associated with lipid metabolism. Conversely, the E17F vs D0F and E17M vs D0M libraries shared 180 common DEGs, primarily involved in melanin metabolism. The clustering heatmap in Fig. 4D showed that the expression patterns of differentially expressed genes in both comparison groups exhibited striking similarity, with females and males distinctly clustered into separate categories at each developmental stage.

### Analysis of GO enrichment in DEGs

**Fig. 5:**
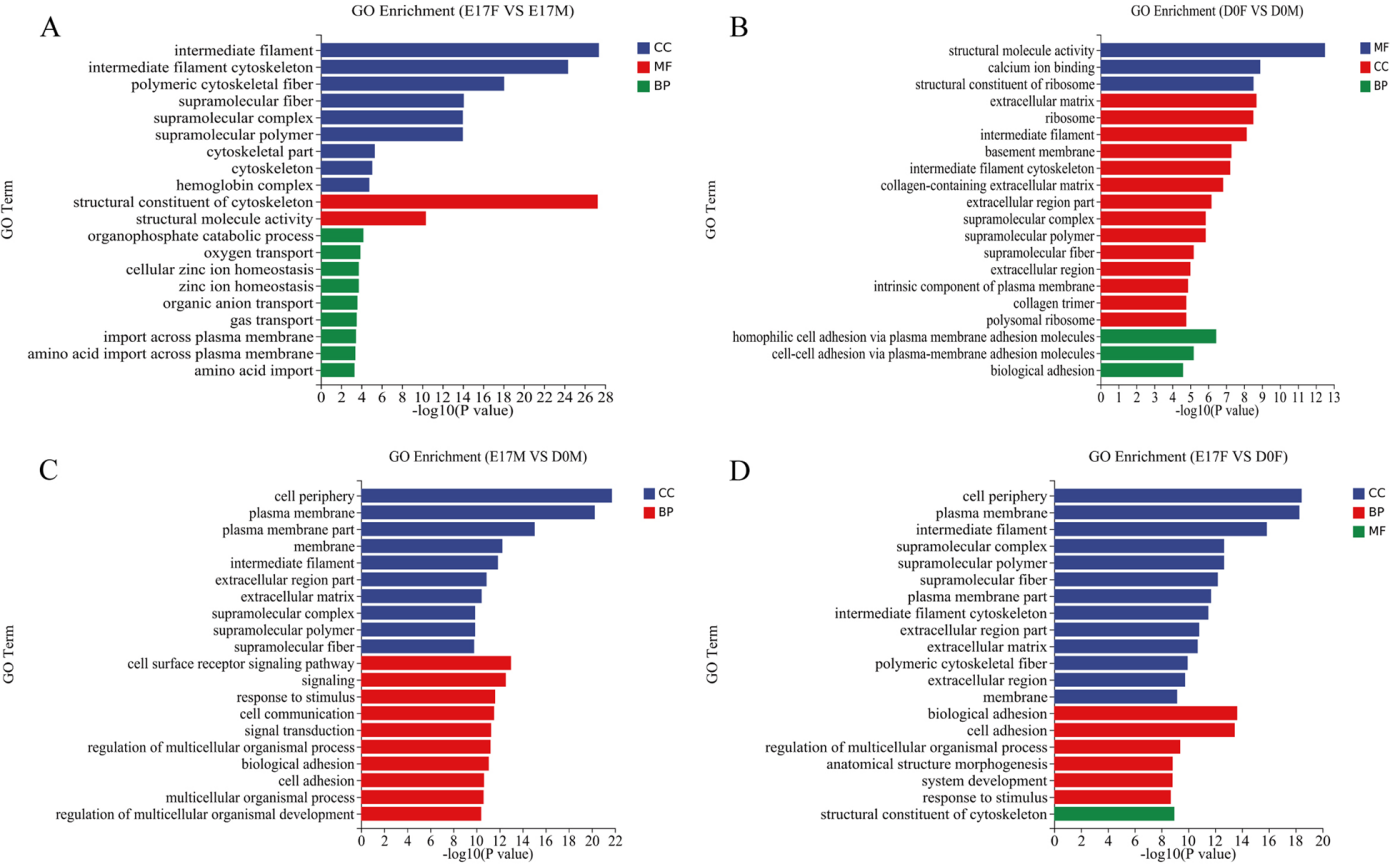
Analysis of GO enrichment in DEGs in dorsal skin tissues of goslings at distinct developmental stages. The GO annotation terms are divided into three main categories: biological processes (BP), cellular components (CC) and molecular functions (MF). The GO classification map of the (**A**) E17F vs. E17M, (**B**) D0F vs. D0M, (**C**) E17M vs. D0M and (**D**) E17F vs. D0F comparisons. (E17F: 17-day female goose embryos, E17M: 17-day male goose embryos, D0F: day-old female goslings, D0M: day-old male goslings)

To gain a comprehensive understanding of the biological implications of DEGs, we conducted a rigorous Gene Ontology (GO) enrichment analysis. The results revealed significant enrichment of GO terms categorized into three fundamental categories: molecular function (MF), cellular component (CC), and biological process (BP). DEGs between E17F and E17M were primarily involved in structural constituent of the cytoskeleton, intermediate filament, and intermediate filament cytoskeleton (Fig. 5A). DEGs between D0F and D0M predominantly demonstrated participation in structural molecule activity, calcium ion binding, and extracellular matrix functions (Fig. 5B). DEGs between E17F and D0F were mainly associated with the cell periphery, plasma membrane, and intermediate filament processes (Fig. 5C). DEGs between E17M and D0M were primarily associated with the cell periphery, plasma membrane, and plasma membrane part functions (Fig. 5D). According to the GO results, three DEGs associated with melanin metabolism were identified between E17F and E17M: ENSACDG00005001594 (solute carrier family 7 member 11), ENSACDG00005014799 (RAB17), and ENSACDG00005003532 (melanocortin 1 receptor). Furthermore, these three DEGs exhibited significant enrichment in processes related to melanin biosynthesis, melanosome transport, and melanosome localization. Notably, a series of DEGs related to melanin and melanosome productionwere significantly downregulated or even silenced in the dorsal skin of D0 goslings compared to those in the dorsal skin of E17 (Fig. 7).

### Analysis of KEGG enrichment in DEGs

**Fig. 6:**
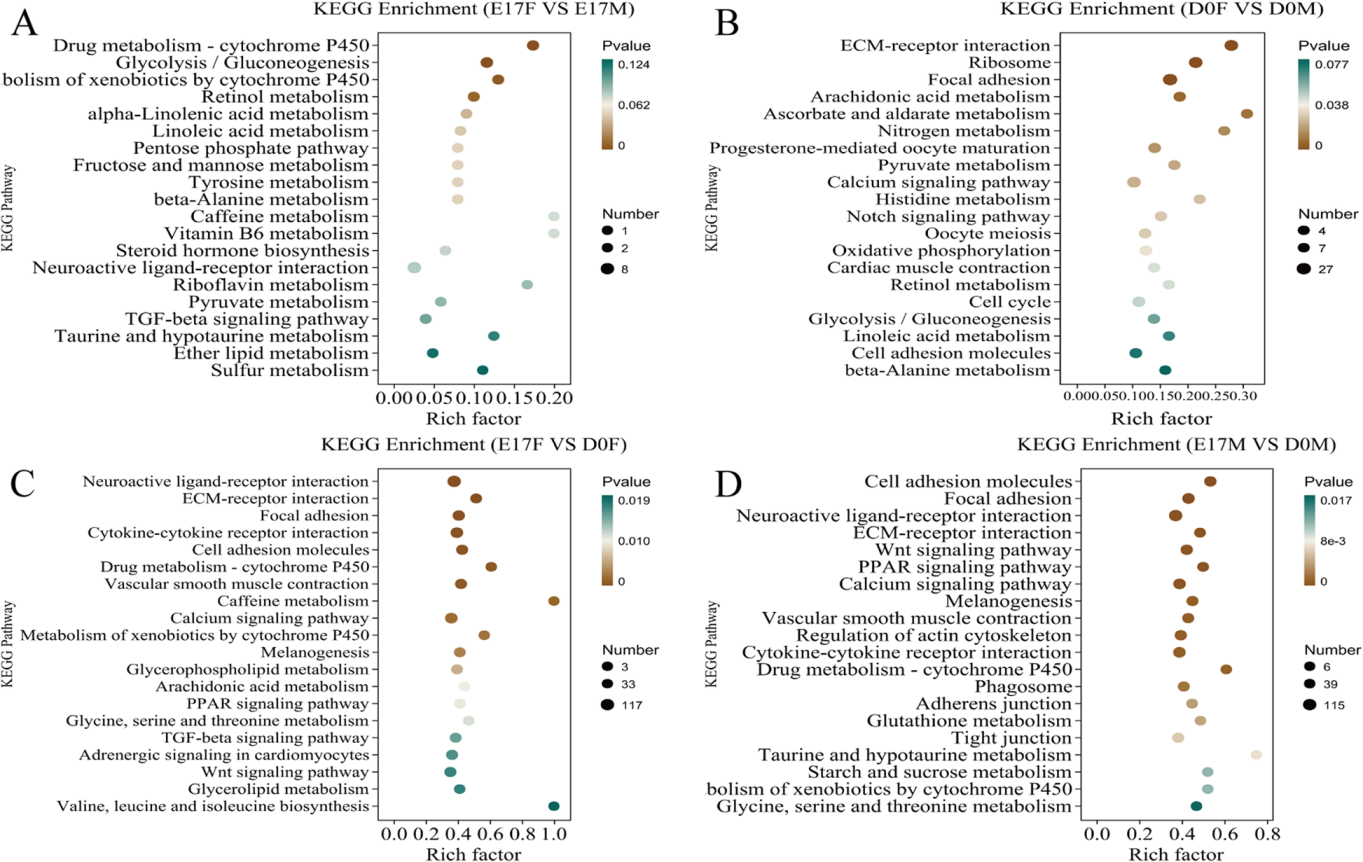
Top 20 enriched KEGG pathways of DEGs in the dorsal skin tissues of goslings at distinct developmental stages. **A** Enriched KEGG pathways in the E17F and E17M groups. **B** Enriched KEGG pathways in the D0F and D0M groups. **C** Enriched KEGG pathways in the E17F and D0F groups. **D** Enriched KEGG pathways in the E17M and D0M groups. (E17F: 17-day female goose embryos, E17M: 17-day male goose embryos, D0F: day-old female goslings, D0M: day-old male goslings)

The DEGs were further analyzed using the Kyoto Encyclopedia of Genes and Genomes (KEGG) database. The top 20 KEGG pathways were categorized into four groups and are shown in Fig. 6. KEGG enrichment analysis revealed “neuroactive ligand receptor interaction” and “glycolysis/gluconeogenesis” as the two most significantly enriched pathways between E17F and E17M (Fig. 6A). In the comparison between D0F and D0M, “focal adhesion” and “ribosome” exhibited the highest enrichment levels among all pathways (Fig. 6B). Furthermore, “neuroactive ligand receptor interaction” and “calcium signaling pathway” were as the two most significantly enriched pathways in both comparisons: E17F vs D0F and E17M vs D0M (Fig. 6C and D).

**Fig. 7:**
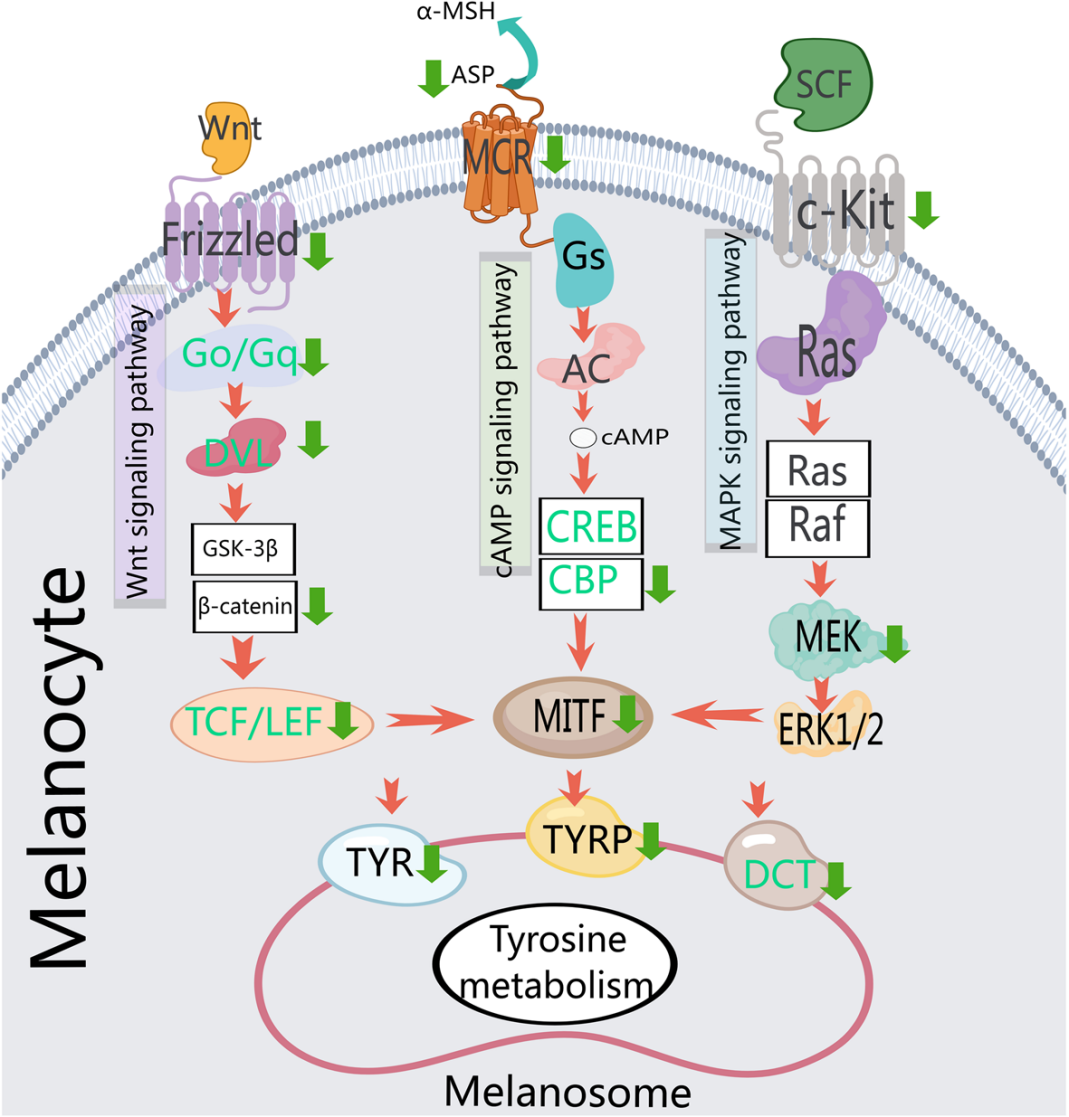
Canonical signaling pathways regulating melanin biosynthesis. A green arrow pointing downward indicates the downregulation of genes in dorsal skin tissues of day-old goslings compared to 17-day-old goose embryos based on transcriptome analysis

The enrichment analysis also revealed significant activation of the "Wnt signaling pathway," "MAPK signaling pathway," and "cAMP signaling pathway", which are involved in melanogenesis and play pivotal roles in regulating feather pigmentation. This was observed in the pairwise comparisons between E17F and D0F, as well as between E17M and D0M. Notably, only one mRNA, ENSACDG00005003532 (melanocortin 1 receptor), was implicated in the melanogenesis pathway when comparing E17F and E17M. Similar to the GO analysis, a series of significant DEGs were observed in the melanogenesis pathway, demonstrating significant downregulation or even silencing in the dorsal skin of D0 goslings compared to that in the dorsal skin of E17 (Fig. 7). Notably, KEGG pathway enrichment analysis also revealed that the largest number of over-represented genes were involved in lipid metabolism and carbohydrate metabolism. A total of 19 and 23 pathways were identified between E17F vs E17M and D0F vs D0M, respectively. These pathways are primarily associated with fatty acid synthesis, phospholipid metabolism, and carbohydrate metabolism. These findings suggested that there are significant differences in lipid and carbohydrate metabolism between male and female goslings at the E17 and D0 developmental stages.

### Expression of genes involved in the melanogenesis process through qRT‒PCR analysis

**Fig. 8:**
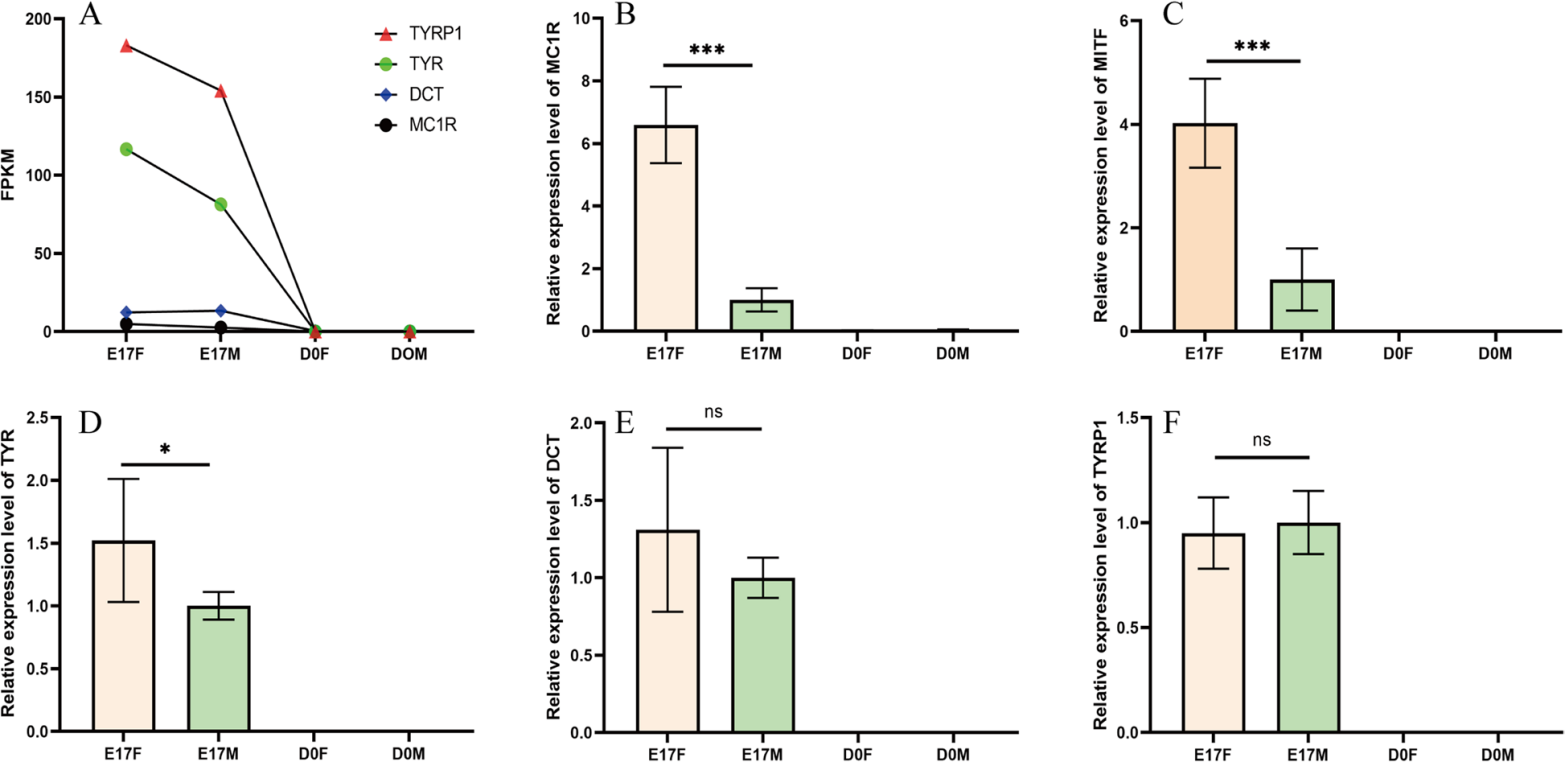
The expression of genes associated with melanogenesis in the dorsal skin tissues of goslings at two distinct developmental stages. **A** represents the expression trends of genes (*TYRP1*, *TYR*, *DCT*, and *MC1R*) from RNA-seq analysis. **B**-**F** show the expression of the *MC1R*, *MITF*, *TYR*, *DCT* and *TYRP1* genes determined via qRT‒PCR. (E17F: 17-day-old female goose embryos, E17M: 17-day-old male goose embryos, D0F: day-old female goslings, D0M: day-old male goslings, *n* = 6; nsp > 0.05, *p < 0.05, ***p < 0.001)

To validate the accuracy of differentially expressed mRNAs identified through transcriptome sequencing technology, we investigated the expression of genes associated with melanogenesis in the dorsal skin tissues of goslings at two developmental stages. We selected four key genes (*MC1R, TYR, DCT,* and *TYRP1*) involved in melanogenesis, which were identified through transcriptome analysis, as candidate genes for qRT‒PCR. The results revealed significant upregulation of *MC1R* gene expression in the dorsal skin of female geese embryos compared to that in the dorsal skin of male geese at the E17 stage (*P* < 0.001) (Fig. 8B). Moreover, there was significantly greater expression of the *TYR* gene in the dorsal skin of female geese than in that of male geese (*P* < 0.05) (Fig. 8D). However, no significant differences were detected in the expression levels of the *DCT* and *TYRP1* genes between the sexes (Fig. 8E and F). All four genes associated with melanogenesis had little or no expression in the dorsal skin tissues of D0 goslings (Fig. 8B and D-F).

Additionally, the observed trend in gene expression across the two developmental stages of gosling dorsal skin tissues was consistent with the findings obtained from the RNA-seq analysis (Fig. 8). Furthermore, the absence of *MITF* in the RNA-Seq data, a crucial player in avian melanogenesis pathway, prompted us to investigate its expression in the dorsal skin tissues of goslings. The results showed that *MITF* expression in the dorsal skin of goslings was significantly greater in female embryos at the E17 stage than in their male counterparts. No detectable expression was found for the D0 goslings (Fig. 8C).

## Discussion

Dorsal coloration in newly hatched Hungarian white geese shows distinct differences between males and females, suggesting the possibility of exploring the genetic mechanisms underlying this trait. In this study, we conducted histological examinations of dorsal skin tissue and observed that compared with males, female geese have more melanin-containing feather follicles and a denser melanin distribution at both the E17 and D0 stages. The results from melanin content measurements using ELISA supported these observations. Our findings are in line with previous studies showing greater melanin deposition in the skin follicles of female geese at different embryonic stages (E14, E18, and E28) [2], indicating a sex-based difference in melanin levels. Similar sexual dimorphism has been observed in other bird species, such as Barn Swallows [6] and mallards [22], suggesting that sex-specific factors influence melanin deposition and feather coloration. Our study and past research indicate that dorsal coloration in newly hatched Hungarian white geese is influenced by melanin quantity and distribution in feather follicles. For example, in domestic rock pigeons, recessive red pigeons have higher pheomelanin and lower eumelanin levels than wild-type blue pigeons [30]. Additionally, yellow plumage in chicken lacks eumelanin [31], suggesting that melanin type affects poultry feather color. Hence, the balance between eumelanin and pheomelanin in the feather follicles on the dorsal skin may contribute to the variations in gosling feather coloration. However, further research is needed for conclusive results.

Both female and male goslings showed a significant decrease in melanin concentrations in the dorsal skin of D0 goslings compared to those of E17. Compared with males, females goslings at D0 exhibited fewer melanin-containing feather follicles in dorsal skin suggesting the absence of melanin in newly formed follicles, resulting in unpigmented feathers that become pure white during adulthood, as observed in white chickens [31].

Melanin, a crucial pigment, greatly influences the coloration of birds [5, 32] and mammals [33]. In our RNA-seq analysis, we identified three DEGs associated with melanin metabolism in E17M compared to E17F. Specifically, solute carrier family 7 member 11 (*SLC7A11*) (ENSACDG00005001594) and *RAB17* (ENSACDG00005014799) were upregulated, while melanocortin 1 receptor (*MC1R*) (ENSACDG00005003532) was downregulated. In mice, the *SLC7A11* gene regulates the pheomelanin pigment in hair and melanocytes, impacting its production with minimal effects on eumelanin [34]. Pheomelanin is responsible for yellow to reddish pigmentation in hair, skin, and eyes [35]. Loss of *SLC7A11* expression inhibits pheomelanogenesis, altering the color of mice from yellow to light cream [34]. Similarly, in rabbits, the expression level of the *SLC7A11* gene was greater in skin with a yellow color than in skin with a black or white color, which was 3.7 times greater than that in skin with a white color [36]. Moreover, *SLC7A11* expression was notably higher in brown alpaca skin than in white skin at both the mRNA and protein levels [37]. Similarly, our findings revealed elevated *SLC7A11* expression in male gosling skin compared to that in female skin. Furthermore, females exhibited darker dorsal plumage, while males displayed faint yellow plumage. This finding suggested that increased *SLC7A11* expression in male gosling skin may enhance melanocyte production of pheomelanin pigment, resulting in faint yellow plumage. In addition, MC1R serves as an α-MSH receptor that plays an important role in α-MSH/cAMP-induced melanogenesis signaling pathways. Studies have shown that mutations and variations in *MC1R* expression are linked to feather color diversity in avian species [9, 11, 38, 39]. Our RNA-seq and qRT‒PCR analyses revealed significant downregulation of the *MC1R* gene in the dorsal skin of male geese at the E17 stage compared to that in the dorsal skin of female geese, suggesting a potential association between *MC1R* and dorsal feather pigmentation in goslings. Previous studies have demonstrated the pivotal role of *MC1R* in regulating avian melanin feather pigmentation [10, 40–45]. Additionally, MC1R is responsive to α-MSH and activates the αMSH/MC1R signaling pathways that stimulate adenylylcyclase (AC), leading to increased intracellular cAMP levels [46, 47]. This activation subsequently activates protein kinase A (PKA) to phosphorylate cAMP response element-binding protein (CREB), facilitating its function as a coactivator in the transcription of MITF [48]. *MITF* levels are regulated via the α-MSH-cAMP-CREB pathway, where elevated cAMP levels increase *MITF* expression and stimulate tyrosinase activity, promoting melanogenesis [49, 50]. Tyrosinase is considered a crucial enzyme that regulates melanin synthesis and facilitates the production of eumelanin by melanocytes [5, 51]. In our study, we observed significant downregulation of two pivotal genes, *AC* and *CREB-binding protein*, in the α-MSH-cAMP-CREB pathway in the dorsal skin of male geese at the E17 stage compared with females. Furthermore, q-PCR analysis revealed consistent expression patterns between the *MITF* and *MC1R* genes, with lower expression levels detected in the dorsal skin of male geese at E17 than in that of female geese. Overall, higher expression levels of *SLC7A11* are associated with increased pheomelanin pigment production, while downregulation of *MC1R*, *AC*, and *CREB-binding protein* inhibits the α-MSH-cAMP-CREB pathway signaling cascade, leading to decreased expression of the *MITF* gene and reduced melanin synthesis. Integrating our histological biochemical findings, RNA-seq, and q-PCR results, the observed sex-specific differences in feather coloration in goslings may be attributed to the differential expression of the *SLC7A11*, *MC1R*, *AC*, *CREB-binding protein* and *MITF* genes. Consequently, females exhibit darker dorsal plumage, while males display faint yellow plumage.Additionally, in our quest to understand the key genes underlying sexual dimorphism in goose feather coloration, RNA-seq analysis revealed a set of significant DEGs associated with three melanogenesis-regulating signaling pathways: α-MSH-cAMP-CREB pathway, Wnt/β-catenin pathway, and mitogen-activated protein kinase (MAPK) pathway. These DEGs exhibited considerable downregulation or complete silencing in the dorsal skin of D0 goslings compared to E17. Consistent with our findings, several previous studies [22, 52–55] have reported similar results from transcriptome and RNA-seq analyses of poultry skin feather follicles. Wnt/β-catenin pathway signaling is extensively involved in and regulates melanocyte development and melanin biosynthesis in avians [56] and mammals [57, 58]. Our RNA-seq analysis revealed a downregulation of key components (*Wnt1*, *Frizzled*, *Go/Gq*, *Dvl*, *β-catenin*, and *TCF/LEF*) involved in the Wnt signaling pathway within the dorsal skin of D0 goslings compared to that of E17. In general, the ligand subtype plays a crucial role in determining the intricate network of Wnt signaling. Our results suggest that the ligand subtype *Wnt1* serves as an activator of the canonical Wnt pathway [59]. Upon binding of Wnt proteins to the transmembrane frizzled (*FZD*) receptor, the canonical signaling pathway is initiated [60], leading to the phosphorylation of disheveled (*DVL*), which directly interacts with *FZD* [61]. Inhibition of the transcriptional activity of the Wnt/β-catenin signaling pathway and T-cell factor/lymphatic enhancer (*TCF/LEF*) can downregulate microphthalmia-associated transcription factor (*MITF*), thereby suppressing melanin synthesis in melanocytes [62]. In addition, we observed a downregulation of key components (*c-Kit* and *MEK*) involved in the MAPK signaling pathway within the dorsal skin of D0 goslings compared to E17. Melanin production is suppressed by inhibiting the mitogen-activated protein kinase (MAPK) signaling pathway, leading to the downregulation of microphthalmia-associated transcription factor (*MITF*) and its downstream targets tyrosinase (*TYR*) and tyrosinase-associated protein (*TYRP*) [63].

In brief, the α-MSH-cAMP-CREB, Wnt/β-catenin, and MAPK signaling pathways are inhibited, resulting in the downregulation of *MITF* expression within the dorsal skin of D0 goslings. This inhibition results in decreased expression of melanogenesis genes such as dopachrome tautomerase (*DCT*), tyrosinase (*TYR*), and tyrosinase-related protein 1 (*TYRP1*) in melanocytes [64–66], as observed in our RNA-seq and qRT‒PCR analyses. A similar study indicated a significant decrease in the expression of these genes from E15 to E28 in geese [67], and these genes became undetectable by E29. Moreover, the TYRP1 mRNA and protein are exclusively expressed in the dorsal skin feather follicles of E18 goslings [2]. These findings suggest a potential association between melanogenesis-related genes and the melanogenic activity of follicular melanocytes (MCs), which are most active during the anagen stage of the feather cycle but become less active or silent during the late feather cycle. In mammals, hair follicle melanogenesis is active only during hair growth, ceases during the catagen stage and is absent during telogen [31, 68]. Our tissue section and biochemical results also revealed a significant decrease in dorsal skin melanin concentrations compared to those in E17, with minimal or no melanin in the feather follicles of D0 goslings.

## Conclusion

The expression of multiple melanogenesis genes determines melanin synthesis in goose feather follicles. The dorsal down coloration of day-old Hungarian white goose goslings shows sexual dimorphism, likely due to differences in the expression of the *MC1R* and *MITF* genes between males and females. The results should help us better understand why male and female goslings have different plumages.

## Data Availability

The data that support the findings of this study are available from the corresponding author upon reasonable request. All transcriptome sequencing reads are openly available in the NCBI SRA at https://submit.ncbi.nlm.nih.gov/, reference number PRJNA1039165.
